# LLM-Augmented Multi-Agent Reinforcement Learning for Cross-Scenario Knowledge Transfer

**DOI:** 10.3390/e28050525

**Published:** 2026-05-06

**Authors:** Chao Li, Yanfei Liu, Jieling Wang, Zhong Wang, Kewei Lu, Chengjin Wang

**Affiliations:** 1 Department of Basic Courses, Rocket Force University of Engineering, Xi’an 710025, China; dalichaoxiao@163.com (C.L.); habaoli520@163.com (J.W.); jesse.ronald@hotmail.com (Z.W.); 17629210720@163.com (K.L.); wangchengjin@nudt.edu.cn (C.W.); 2 Department of Optical Communication Networks, Information Support Force Engineering University, Wuhan 430035, China

**Keywords:** multi-agent reinforcement learning, large language models, low-rank adaptation, knowledge transfer, dynamic prompt, annealing Kullback–Leibler divergence

## Abstract

Multi-agent reinforcement learning (MARL) relies on trial-and-error interactions to update policies. However, trial-and-error learning typically requires extensive interactions to achieve satisfactory performance, resulting in low sample efficiency, which limits its application in the real world. To reduce the trial-and-error costs of MARL and accelerate the convergence of multi-agent collaborative policies, we propose a MARL policy transfer method named LoLM-MARL, based on fine-tuning large language models (LLMs). First, leveraging the general world knowledge and reasoning capabilities of LLMs, low-rank adaptation (LoRA) is employed to fine-tune the pre-trained model on source tasks, thereby providing general decision-making knowledge for cross-scenario policy transfer. Second, a dynamic prompt construction method for LLMs is designed. By dynamically eliminating the state information of ineffective agents from the prompts, the method provides denser observation data for the large language model, thereby enhancing its policy representation capability in specific complex collaborative scenarios. Meanwhile, the dynamic prompt design concept enriches the training sub-scenarios for the algorithm, thereby laying the foundation for the model to learn more general decision-making knowledge. Finally, a Kullback–Leibler (KL) divergence regularization method based on an annealing strategy is constructed to ensure consistency between the policy distributions of the fine-tuned model and the pre-trained model, effectively mitigating the catastrophic forgetting problem during the fine-tuning process of the pre-trained model. Experimental results show that in zero-shot transfer tasks, LoLM-MARL achieves a maximum improvement of 101.4% in average win rate compared to existing state-of-the-art (SOTA) methods. In six few-shot transfer tasks, our method consistently achieves better generalization performance than traditional SOTA methods, and improves the convergence speed by 4 to 30 times compared to the training-from-scratch approach, providing a new solution paradigm for efficient policy transfer in complex dynamic environments.

## 1. Introduction

Multi-agent reinforcement learning (MARL), drawing inspiration from human trial-and-error exploration mechanisms, has demonstrated significant potential in addressing complex multi-agent decision-making problems [1]. In recent years, it has been widely applied in fields such as embodied intelligence [2], autonomous driving [3], and distributed systems [4]. Despite important progress in MARL, it still faces numerous challenges. Among these, the low sample utilization efficiency of algorithms is a core challenge [5], primarily reflected in the following: reinforcement learning relies on continuous trial-and-error to obtain environmental feedback signals, which are then used to guide policy updates. However, trial-and-error learning, without the guidance of prior knowledge, requires vast amounts of interactions to learn a satisfactory optimal policy, resulting in generally low exploration efficiency and sample utilization. This phenomenon becomes more pronounced in environments with sparse rewards [6], high-dimensional state spaces [7], and non-stationary environments [8], severely constraining the training quality and convergence efficiency of algorithms while increasing deployment costs and difficulties in practical applications.

To improve the sample efficiency of algorithms, that is, to reduce the amount of environment interaction data required for the algorithm to achieve threshold performance, researchers have proposed a series of targeted measures in recent years, among which knowledge reuse methods have received widespread attention [9,10]. Based on the concept of transfer learning, this approach applies the optimal collaborative policies learned by the algorithm in source tasks to unseen target tasks, enabling the algorithm to fully leverage previously acquired experience and policies for rapid initialization in new tasks, thereby reducing training costs. A core challenge in studying cross-scenario transfer for MARL lies in ensuring the effectiveness of knowledge transfer from source to target tasks. Currently, existing methods addressing the issue of effective knowledge transfer primarily focus on three aspects: online multi-task representation learning [11,12,13,14], offline multi-task universal skill learning [15,16,17], and universal subtask decomposition [18]. Online multi-task representation learning trains multiple source tasks in parallel to learn cross-task general knowledge, thereby improving the algorithm’s performance on a single unseen task. While this method helps enhance sample utilization efficiency and transfer performance, it also suffers from issues such as gradient conflicts [14] and training imbalance [11,14] caused by differences between tasks. Furthermore, online multi-task representation learning requires frequent interactions with the environment to simultaneously learn or fine-tune policies for different tasks, a process that is typically costly. In contrast, offline multi-task universal skill learning can learn more general collaborative skills only relying on static multi-task offline data, effectively reducing the computational and time overhead of online representation learning. Nevertheless, its performance highly depends on the quality of the training dataset. When the dataset lacks sufficient optimal trajectories or diversity, agents struggle to learn general skills and optimal policies from source tasks, which further limits their adaptability in new tasks. Different from multi-task MARL, the universal subtask decomposition method decomposes source tasks into several task-independent subtasks and endows them with cross-task general semantics to achieve effective knowledge transfer in target tasks. However, when facing complex situations such as significant task differences or high task diversity, the extracted “task-independent” subtasks often fail to meet the policy requirements of target tasks, thereby impairing the effectiveness of cross-task knowledge transfer.

In recent years, large language models (LLMs) have played a crucial role in various fields, including energy [19], medical [20], chemistry [21], and robot control [22], owing to their extensive world knowledge and powerful reasoning capabilities. Currently, several studies have combined LLMs with reinforcement learning (RL) and achieved preliminary results [23,24,25], yet the cross-disciplinary exploration of LLMs and MARL remains relatively limited. Inspired by this, this paper proposes a cross-scenario transfer method for MARL based on LLMs, utilizing the robust semantic understanding and task-reasoning capabilities of LLMs along with the low-rank adaptation (LoRA) [26] fine-tuning technique. This method employs the lightweight LoRA fine-tuning technique to preserve the general knowledge of the pre-trained model while enabling training on specific decision-making tasks and efficient cross-scenario transfer through fine-tuning low-rank parameter matrices on a small scale. It aims to address the performance bottlenecks of traditional MARL in policy transfer across complex scenarios, with the core objective of enhancing the cross-scenario generalization ability of the algorithm.

However, adapting LLMs to MARL primarily faces the following two challenges: (1) Although LLMs possess extensive prior knowledge, the type of knowledge may not be well-adapted to specific cross-scenario transfer decision-making tasks, thereby limiting their generalization ability across scenarios; and (2) during the fine-tuning process for complex decision-making tasks, LLMs often suffer from catastrophic forgetting. Even when only small-scale updates are made to the low-rank adaptation matrix parameters via LoRA, the model still cannot fundamentally avoid forgetting the existing knowledge of the frozen pre-trained model, thereby affecting the algorithm’s stable convergence in the source task. To address these challenges, the main contributions and solutions of this paper are as follows:

(1) To address the insufficient generalization of traditional MARL in cross-scenario transfer, we propose LoLM-MARL, a cross-scenario transfer method for MARL based on lightweight LoRA fine-tuning of large language models. It establishes a semantics-driven multi-agent collaborative decision-making mechanism in complex dynamic environments, representing an exploratory effort to enhance cross-scenario generalization in MARL with the assistance of LLMs.

(2) A dynamic prompt design scheme for LLMs is proposed to enhance LLMs’ feature extraction capability and generalization. This scheme dynamically eliminates redundant information in prompts, providing an LLM with state information of higher density to improve its semantic understanding and feature extraction effectiveness. Furthermore, this design naturally exposes the LLM to more diverse training scenarios during fine-tuning, facilitating the adaptation of general world knowledge to specific decision-making tasks and laying the foundation for zero-shot or few-shot policy transfer.

(3) An annealing-strategy-based KL divergence regularization method is proposed to address the catastrophic forgetting problem during LLM fine-tuning. This method constrains the action probability distributions between the pre-trained LLM and the fine-tuned LLM via KL divergence, ensuring that the fine-tuned model maintains a consistent action distribution with the pre-trained model in the early stage of policy exploration, thereby promoting stable convergence of LoLM-MARL. Meanwhile, the annealing strategy dynamically adjusts the constraint strength to flexibly balance policy exploration and exploitation.

## 2. Related Work

### 2.1. Generalization Challenges in MARL

When tackling complex multi-agent sequential decision-making problems, cross-scenario generalization ability is crucial [27]. This ability not only serves as a prerequisite for efficient exploration and high sample efficiency but also significantly reduces the cost of policy transfer, providing strong support for the practical deployment and engineering applications of MARL. For this reason, determining how to effectively enhance the generalization performance of MARL has gradually attracted increasing attention from scholars in recent years.

Currently, existing works mainly focus on two aspects: multi-task learning and subtask decomposition. Multi-task multi-agent reinforcement learning (MT-MARL) improves sample efficiency and transferability by training agents to simultaneously learn multiple related tasks. Depending on the mode of policy learning, MT-MARL can be categorized into online learning and offline training. Wang et al. [11] employed online multi-task pre-training to learn general cooperative policies. The core idea involves decoupling the perception and decision-making modules and extracting shared decision logic from similar tasks to enable fast adaptation to new tasks. Li et al. [12] explicitly modeled inter-agent dependencies via interactive value decomposition and utilized local trajectories of each agent to learn task representations for effectively evaluating task similarity, thereby achieving targeted cross-task knowledge transfer. Liang et al. [13] proposed a two-stage curriculum learning framework that refines policies learned in previous curricula into general policies through a gradient-based adaptive meta-learning algorithm, thus effectively alleviating the persistent adaptation problem in MARL multi-task training. Zhu et al. [14] tackled task difficulty disparity and imbalanced training in online multi-task learning by using pre-trained language models to encode task semantics. They employed Transformers to dynamically integrate observations with task representations and introduced a task-regret-based adaptive weighting mechanism, enabling efficient parallel training and cross-scenario generalization. Furthermore, Zhu et al. [28] addressed policy transfer across unrelated tasks with a hierarchical multi-task learning framework based on a skill graph. Their method comprises a high-level module for skill graph construction, selection, and combination via a scoring mechanism, and a low-level module for policy execution. This approach effectively enhances knowledge generalization across unrelated tasks. Although online multi-task learning plays an important role in enhancing the generalization of MARL, such methods inevitably incur high training costs and tend to suffer from gradient conflicts and training imbalance when task differences are substantial.

To overcome the inherent limitations of online MT-MARL, Zhang et al. [15] proposed an offline MT-MARL method, which learns task-agnostic general skills solely from offline datasets of multiple tasks and selects optimal skills under the centralized training with decentralized execution paradigm, finally achieving promising zero-shot generalization on unseen target tasks. To further improve generalization on unseen tasks, Liu et al. [16] proposed a hierarchical decoupled skill discovery framework. This framework learns effective skill representations from limited multi-task offline data by jointly learning general task skills and task-specific skills in parallel. Although offline MT-MARL substantially reduces training costs, its asymptotic performance in source tasks heavily depends on the quality of the offline data [29]. If the coverage of multi-agent trajectories is insufficient or lacks adequate optimal trajectories, agents will struggle to learn general decision-making skills, thereby affecting cross-scenario generalization ability.

Different from the above two approaches, Tian et al. [18] explored an alternative approach by decomposing source tasks into a series of task-agnostic general subtasks. Through a scalable subtask encoder and an adaptive semantic module, they effectively ensured the cross-scenario semantic consistency of subtasks, thereby achieving effective cross-scenario transfer. However, when facing complex tasks or tasks with significant differences, the adaptability of these subtasks is limited, consequently reducing cross-scenario transfer performance.

### 2.2. LLMs for Decision-Making

In recent years, with the rapid development of LLMs, they have shown great potential in decision-making tasks that require complex scenario understanding and reasoning capabilities [30,31]. Their powerful semantic understanding ability can more accurately understand the dependencies between agents and dynamic decision-making scenarios than traditional models. However, most existing studies focus on LLM-assisted single-agent reinforcement learning, and there is still a lack of systematic study on how to effectively leverage the advanced semantic understanding and reasoning abilities of LLMs in MARL. Li et al. [32] investigated sample efficiency and generalization in autonomous driving tasks, proposing an LLM-based hierarchical reinforcement learning framework. In this framework, the LLM serves as a high-level planner, providing semantic understanding and long-term goals. The low level promotes efficient policy learning through goal-conditioned skill transfer, and its generalization capability in unseen scenarios was validated within an autonomous driving simulation system. Similarly, reference [33] leveraged LLMs to enhance the performance of hierarchical reinforcement learning in complex long-horizon sequential decision-making tasks. This approach employs the LLM as a teacher agent to guide exploration in high-level policy, while the low-level adopts a policy library-based selective execution mechanism. The synergistic collaboration between the two effectively addresses the exploration challenge in long-horizon decision-making under sparse rewards. Zhou et al. [34] addressed path planning for heterogeneous agents by integrating the local exploration advantages of Q-learning with the global understanding capability of LLMs for unknown environments. By designing a problem-modeling-oriented prompting strategy, they transformed complex scheduling tasks into semantic descriptions understandable by LLMs, significantly improving path planning efficiency and algorithm convergence. Zhang et al. [35] utilized the reasoning and analysis capabilities of LLMs to optimize the iterative process of RL, effectively solving the joint optimization problem of energy cost control and carbon emission reduction in energy systems. Wang et al. [36] introduced LLMs to optimize the training process of RL and neural networks, achieving high-precision target tracking control of a bionic robotic arm. Dalal et al. [37] used LLMs to generate high-level expert motion plans, decompose complex tasks into phased subgoals, and guide a single reinforcement learning policy to execute step-by-step. This method significantly improves the ability of robots to complete long-horizon tasks and achieves a success rate of over 85% across benchmark tests.

In summary, existing research demonstrates that integrating LLMs with RL, particularly within hierarchical reinforcement learning architectures, has become an effective paradigm for addressing key challenges such as long-horizon decision-making, sparse rewards, and cross-scenario generalization in single-agent settings. However, current successful applications remain largely confined to single-agent scenarios. The rich world knowledge and powerful reasoning capabilities inherent in LLMs also hold significant potential to enable efficient learning and generalization for MARL in complex decision-making environments. Inspired by this potential, this paper introduces LLMs into the domain of MARL, focusing specifically on addressing the challenge of limited cross-scenario generalization in traditional MARL. This work represents an exploratory effort at the intersection of LLMs and MARL.

## 3. Preliminaries

### 3.1. Dec-POMDPs

Multi-agent reinforcement learning can be viewed as a decentralized partially observable Markov decision process (Dec-POMDPs) [38], typically defined by $\langle S,A,T,R,O,n,\gamma \rangle$. S is the state space, A is the joint action space, $T:S\times A\times S^{\prime }\to \left[0,1\right]$ is the state transition probability, representing the probability of transitioning from state S to the next state $S^{\prime }$ given the joint action $A=\left(a_{1},\ldots ,a_{n}\right)$. $R:S\times A\times S^{\prime }\to R$ denotes the instant reward after transitioning from state S to state $S^{\prime }$ following the joint action. O is the set of local observations for all agents in state S, n is the number of agents, and $\gamma \in \left[0,1\right]$ is the discount factor used to balance instant rewards and future returns. At each decision time step, each agent i selects an action $a_{i}$ based on its local observation $o_{i}$ and its policy $\pi_{\theta }(a_{i}|o_{i})$, forming a joint action $a\in A_{n}$. The joint action transitions the multi-agent system to the next state $S^{\prime }$ according to the state transition function T. The environment then provides an immediate reward $r\in R\left(s,a\right)$ to the agents based on the joint action. Each agent learns a policy to maximize the future discounted cumulative reward $J\left(\theta \right)=E\left[\sum_{t=0}^{\infty }\gamma^{t}R\left(s^{t},a^{t}\right)\right]$.

### 3.2. Multi-Agent Proximal Policy Optimization (MAPPO)

Multi-Agent Proximal Policy Optimization (MAPPO) [39] is the multi-agent extension of the PPO. Its strong performance, simplicity, and excellent compatibility with the centralized training with decentralized execution (CTDE) framework have made it widely adopted in MARL research. Based on the Actor–Critic framework, MAPPO trains individual policy networks for each agent while sharing a common value network across all agents. During policy learning, the objective is to maximize the following function:

(1) $$L\left(\theta \right)=\left[\frac{1}{Bn}\sum_{i=1}^{B}\sum_{k=1}^{n}\min\left(r_{\theta ,i}^{\left(k\right)}A_{i}^{\left(k\right)},clip\left(r_{\theta ,i}^{\left(k\right)},1-\epsilon ,1+\epsilon \right)A_{i}^{\left(k\right)}\right)\right]+\sigma \frac{1}{Bn}\sum_{i=1}^{B}\sum_{k=1}^{n}S[\pi_{\theta }(o_{i}^{\left(k\right)})]$$

where $r_{\theta ,i}^{\left(k\right)}=\pi_{\theta }(a_{i}^{\left(k\right)}|o_{i}^{\left(k\right)})/\pi_{\theta_{old}}(a_{i}^{\left(k\right)}|o_{i}^{\left(k\right)})$, $A_{i}^{\left(k\right)}$ denotes the advantage function, S represents the policy entropy, σ is the entropy coefficient hyperparameter, and B denotes the batch size.

The value network is updated by minimizing the following function:

(2) $$L(\phi )=\frac{1}{Bn}\sum_{i=1}^{B}\sum_{k=1}^{n}max[C,D]$$

where $C=V_{\phi }{\left(s_{i}^{\left(k\right)}-{\hat{R}}_{i}\right)}^{2}$, $D={\left(clip(V_{\phi }(s_{i}^{\left(k\right)}),V_{\phi_{old}}(s_{i}^{\left(k\right)})-\varepsilon ,V_{\phi_{old}}(s_{i}^{\left(k\right)})+\varepsilon )-{\hat{R}}_{i}\right)}^{2}$, ${\hat{R}}_{i}$ denotes the cumulative discounted reward.

### 3.3. Low-Rank Adaptation (LoRA)

Low-rank adaptation (LoRA) [26], as a mainstream parameter-efficient fine-tuning method, is inspired by the findings of Aghajanyan et al. [40]: while the weight matrices of pre-trained models are full-rank, weight updates during task-specific adaptation often exhibit low-rank characteristics. Building on this insight, LoRA freezes the original pre-trained weights and decomposes the weight update matrix into two low-rank parameter matrices. By updating only these two low-dimensional matrices, LoRA enables efficient fine-tuning in target domains. Its mathematical principle is as follows:

For a pre-trained weight matrix $W_{0}\in R^{d\times k}$, it is kept frozen during fine-tuning on downstream tasks, while the weight update matrix ΔW is decomposed into the product of two low-rank matrices $A\in R^{r\times k}$ and $B\in R^{d\times r}$:

(3) ΔW=BA

And then, the updated model weight becomes:

(4) $$W=W_{0}+\Delta W=W_{0}+BA$$

During forward propagation, assuming the input is x, the model output after incorporating the low-rank parameter matrices can be expressed as:

(5) $$h=W_{0}x+\Delta Wx=W_{0}x+BAx$$

**Figure 1:**
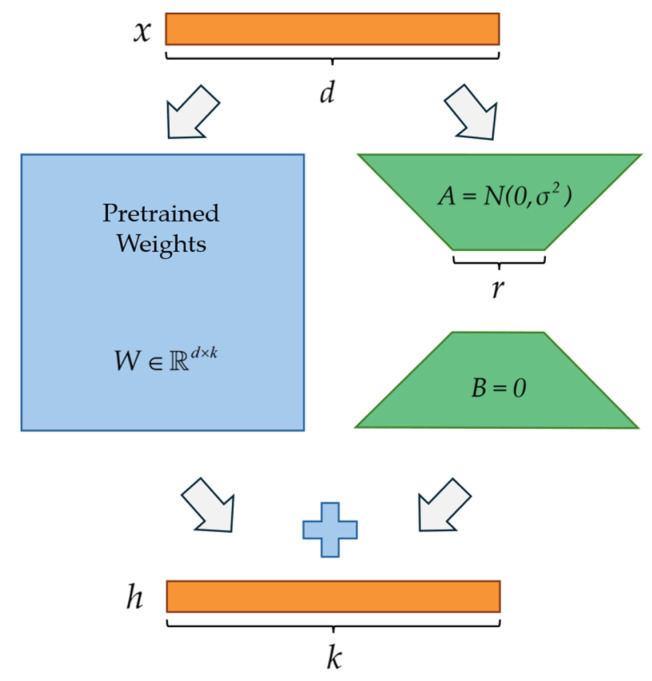
LoRA technical framework.

Figure 1 shows the framework of LoRA. Here, matrix A is initialized with a random gaussian distribution, and matrix B is initialized as a zero matrix to ensure consistency between the model behavior and the initial performance of the pre-trained model. During forward propagation, the input x is fed in parallel into the frozen original weight branch and the trainable low-rank weight branch. The final output $h=W_{0}x+BAx$ is the sum of the original output and the low-rank updated output.

## 4. Methodology

In this section, we will provide a detailed introduction to the LoLM-MARL, which primarily comprises the following three components: (1) dynamic prompt construction for LLM; (2) mitigation of catastrophic forgetting in the LLM during fine-tuning; and (3) network architecture design for adaptive agent numbers in the transfer process. Accordingly, the section begins with an overview of the overall algorithm framework, followed by a detailed exposition of the specific implementation details for each component.

### 4.1. Overview of LoLM-MARL

**Figure 2:**
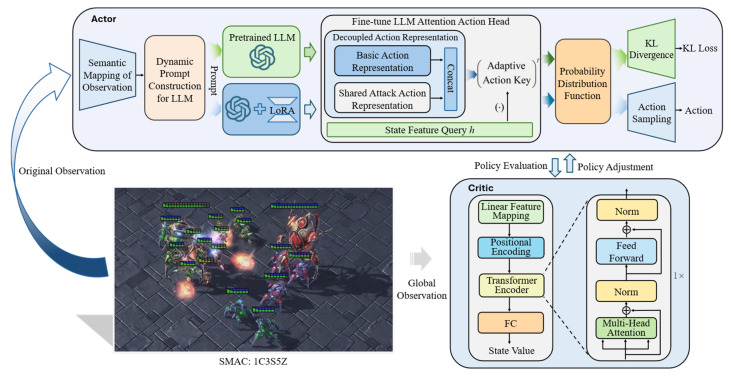
Overall framework of LoLM-MARL.

The overall framework of LoLM-MARL is shown in Figure 2. The algorithm mainly consists of two components: the LLM policy network fine-tuned via LoRA, and the value network based on the Transformer. For the policy network, we first perform semantic mapping on the raw numerical observation matrix and construct dynamic LLM input according to the prompt template. To ensure training stability, the hidden state features of both the frozen pre-trained LLM and the fine-tuned LLM are fed into the shared-attention action head of the fine-tuned LLM. The action probability distributions of the two are constrained by KL divergence with an annealing strategy to alleviate catastrophic forgetting during fine-tuning. The final action output of the policy network is generated by the LoRA-fine-tuned LLM, while the frozen pre-trained LLM only contributes to the KL loss calculation and does not directly participate in action generation. Furthermore, to enable effective adaptation to dynamic changes in the number of agents during cross-scenario transfer, we have designed specialized structures for both the policy network and the value network. Detailed implementation specifics are provided in Section 4.4.

### 4.2. Dynamic Prompt Design for LLM

In reinforcement learning, the data is derived from the agent’s observations of the interactive environment, and such observations are typically represented as structured numerical tensors, whereas LLM-based reinforcement learning requires semantically rich natural language sequences as input. To bridge this gap, this section introduces a customized dynamic prompt framework for the StarCraft Multi-Agent Challenge (SMAC) environment. To enhance the cross-scenario transferability of the fine-tuned model, we dynamically filter out information of deceased agents from the prompts, thereby reducing irrelevant state information that could interfere with LLMs’ decision-making. This dynamic processing naturally covers various types of multi-agent adversarial scenarios, ensuring the algorithm’s transfer capability across different environments. Furthermore, the adaptive adjustment of prompt sequence length reduces the time required for tokenization and forward inference in the LLM, thereby improving the overall efficiency of model fine-tuning.

Before constructing the LLM prompts, the raw numerical observation matrix must first undergo semantic mapping. To illustrate the transformation more clearly, Table 1 and Table 2 detail the composition of agent observations and actions in SMAC, respectively.

**Figure 3:**
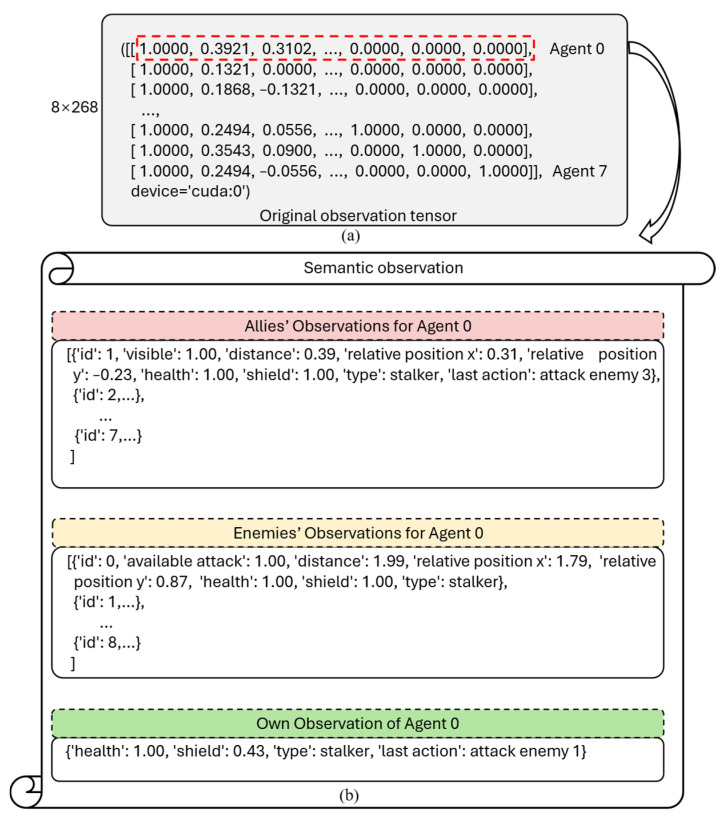
Form changes in agent 0′s observation data before and after mapping. (**a**) original data format; (**b**) mapped data format.

Taking the 3s5z_vs_3s6z scenario as an example, Figure 3 illustrates the format transformation of the observation data for agent 0 before and after mapping. Figure 3a shows the observation matrix of 8 agents, where each row vector integrates observation information, including allies, enemies, and the agent itself. Each position in the observation vector is composed of elements listed in Table 1 and Table 2. Among them, numerical values are uniformly rounded to two decimal places, and the entity data mapping follows the order in the original observation vector (allies → enemies → self). By mapping the raw observation information of all agents into the natural language descriptions shown in Figure 3b, the observation data becomes more semantically expressive, laying the foundation for the subsequent construction of dynamic prompts.

**Figure 4:**
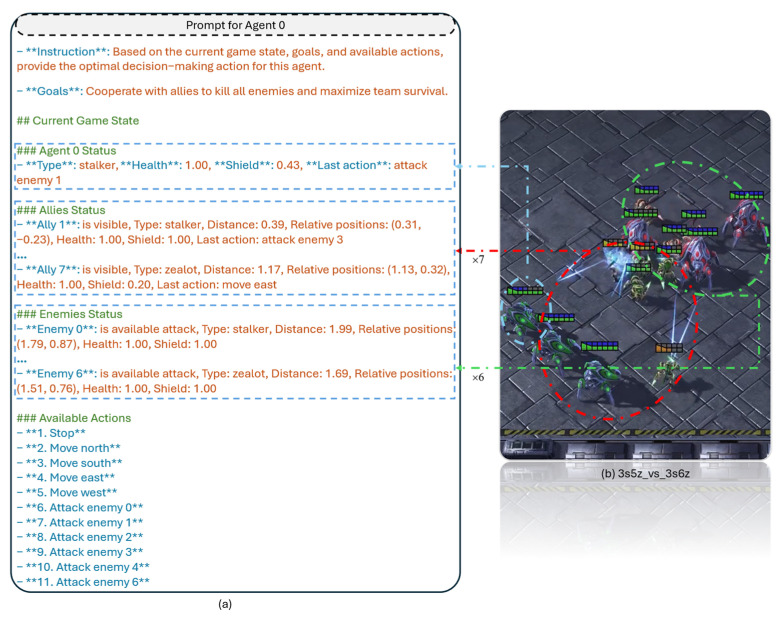
Design of dynamic prompts for agent 0 in 3s5z_vs_3s6z map. (**a**) the composition of agent 0’s prompt; (**b**) current battlefield situation at this timestep.

Based on the above semantic observation data, we proceed to construct the LLM prompt. To provide the LLM with denser prompt information, we adopt a dynamic prompt construction method to timely remove the observation information of dead agents, thereby reducing interference with its decision-making. Moreover, the dynamic prompt design naturally covers more adversarial training scenarios, thus ensuring the transfer performance of the fine-tuned model and promoting the generalization of the algorithm across different scenarios. Figure 4 illustrates the design idea of dynamic prompt. Figure 4a shows the prompt for agent 0 in the 3s5z_vs_3s6z scenario, which consists of four components: LLM instruction, goal, current game state, and available actions. The LLM instruction specifies the functional requirements and constraints for the model; the goal defines the long-term and short-term tasks of the agent; the current game state includes observations of the agent itself, allied units, and enemy units. As shown in Figure 4b, the enemy formation consists of 6 combat units (the green circle), corresponding to only 6 semantic observation entries for enemy units in the left figure. Similarly, it can be seen from the available actions that there are 6 attackable agents at present. The available actions cover all valid actions available to agent 0 at the current timestep. Figure 4 only shows part of the prompt information for agent 0. For detailed information, see Appendix A.

### 4.3. Policy Annealing Alignment for Pre-Trained and Fine-Tuned LLMs

During the fine-tuning of LLMs, catastrophic forgetting is commonly observed. Although LoRA achieves efficient task-specific adaptation by freezing the pre-trained parameters of LLMs and only training low-rank adaptation matrices, the model still cannot fundamentally avoid catastrophic forgetting when handling complex decision-making tasks, which adversely affects the stable convergence of the algorithm. To address this issue, this section adopts the KL divergence regularization method to constrain the action probability distributions of the frozen pre-trained model and the fine-tuned model. Additionally, an annealing strategy is introduced to dynamically adjust the regularization strength, effectively balancing exploration and exploitation of the policy. The specific implementation details are as follows.

The loss function of the LoLM-MARL policy network is shown in Equation (6), which consists of two components: the policy loss of MAPPO and the KL divergence loss:

(6) $$L_{total}(\theta )=L_{MAPPO}(\theta )+\lambda \cdot L_{KL}(\theta )$$

Here, $L_{MAPPO}(\theta )$ consists of two terms: the clipped policy loss and the entropy regularization loss, as shown in Equation (7), and $L_{KL}(\theta )$ is given by Equation (8):

(7) $$L_{MAPPO}\left(\theta \right)=\left[-\frac{1}{Bn}\sum_{i=1}^{B}\sum_{k=1}^{n}\min\left(r_{\theta ,i}^{\left(k\right)}A_{i}^{\left(k\right)},clip\left(r_{\theta ,i}^{\left(k\right)},1-\epsilon ,1+\epsilon \right)A_{i}^{\left(k\right)}\right)\right]-\sigma \frac{1}{Bn}\sum_{i=1}^{B}\sum_{k=1}^{n}S[\pi_{\theta }(o_{i}^{\left(k\right)})]$$

(8) $$\begin{array}{l} L_{KL}(\theta )=\frac{1}{Bn}\sum_{i=1}^{B}\sum_{k=1}^{n}D_{KL}\left(\underset{Frozen\;LLM}{\underbrace{\pi_{\theta_{0}}(o_{i}^{\left(k\right)})}}∥\underset{LoRA\;LLM}{\underbrace{\pi_{\theta }(o_{i}^{\left(k\right)})}}\right)\\ \;\;\;\;=\frac{1}{Bn}\sum_{i=1}^{B}\sum_{k=1}^{n}\pi_{\theta_{0}}(o_{i}^{\left(k\right)})log\frac{\pi_{\theta_{0}}(o_{i}^{\left(k\right)})}{\pi_{\theta }(o_{i}^{\left(k\right)})} \end{array}$$

where B is the batch size, n is the number of agents, and σ is the entropy coefficient hyperparameter.

Unlike typical KL divergence designs, to ensure the diversity of policy learning in the fine-tuned model, this paper adopts an annealing design for the regularization parameter λ of KL divergence. Its core idea is to dynamically adjust the constraint strength imposed by the frozen LLM on the fine-tuned LLM at different stages of policy learning, thereby balancing alignment between the two policies and the diversity of exploration. In the initial learning stage, due to the high randomness of the model’s exploration mechanism, a stronger constraint should be applied. As the network is continuously updated and the model gradually learns the optimal policy, the constraint strength should decay gradually with the training timesteps. The detailed parameter design of λ is given as follows:

(9) $$\lambda_{t}=\left\{\begin{array}{l} \lambda_{0}-\frac{\lambda_{0}}{t_{1}}*t\;if\;t<t_{1}\\ \;\;\;\;0\;\;\;\;else \end{array}\right.$$

(10) $$\lambda_{0}=\frac{L_{MAPPO}(\theta )}{L_{KL}(\theta )}$$

At the beginning of training, to balance the MAPPO loss and the KL divergence loss and avoid a significant gap between them, $\lambda_{0}$ is set according to Equation (10). As the timestep t increases, λ decreases linearly at a decay rate $\lambda_{0}/t_{1}$ until it reaches zero at training step $t_{1}$. Here, $t_{1}$ is a manually tuned hyperparameter. It should be noted that the KL divergence loss is only applied during the LoRA fine-tuning and is not involved in either few-shot transfer or zero-shot transfer.

Since LoRA is initialized to zero, the resulting issue is that the KL divergence loss may be close to zero at the beginning of training, which makes Equation (10) numerically unstable. To ensure the stability of algorithm training during the early stage of policy exploration, we adopt the following safeguard mechanism to avoid the aforementioned problem. First, we apply smoothing to the denominator of Equation (10) by using $\max(\mathrm{KL},1\times 10^{-4})$ as the denominator to avoid numerical instability caused by division by zero. Second, based on the loss values obtained from multiple cross-validations of $L_{KL}(\theta )$ and $L_{MAPPO}(\theta )$, we reasonably set the hyperparameter $\lambda_{0}$ to avoid gradient explosion caused by an excessively large $\lambda_{0}$ resulting from over-pursuing the balance between $L_{KL}(\theta )$ and $L_{MAPPO}(\theta )$.

### 4.4. Adaptive-Scale Transfer Network Architecture

Cross-scenario transfer in MARL inevitably faces challenges caused by dynamic changes in the number of agents. Specifically, deep reinforcement learning approximates the optimal policy via neural networks, whose weight dimensions are usually fixed, resulting in mismatches between input–output dimensions and network weights during transfer. In response to these challenges, this section will focus on the targeted design of policy network and value network to achieve flexible cross-scenario transfer.

For the policy network, state feature extraction relies on the LLM. The Transformer architecture [41] of the LLM inherently supports the processing of variable-length sequence inputs. Its core parameter matrix dimensions are only related to the hidden feature dimension and are completely decoupled from sequence length. Changes in the number of agents only affect the size of intermediate computation matrices rather than the network structure itself. However, for the action head of the policy network, the action output dimension of the traditional linear layer is related to the number of agents and remains fixed. To enable the feature dimension of the action head to dynamically adapt to variations in the number of agents, this section proposes an attention action head by considering the characteristics of the interactive environment. Specifically, we decouple the action head into two components: a base action head and an attack action head (as shown in Figure 2). The action head takes the hidden state $h\in R^{d}$ output by the LLM as input, where d is the hidden dimension of the LLM, and uses this hidden state as the state feature query for the action head. The action head maintains two learnable parameter matrices: a base action embedding $W_{\mathrm{base}}\in R^{6\times d}$, corresponding to six fixed actions (no operation, stop, move north, move south, move west, move east), and an attack action embedding $W_{\mathrm{attack}}\in R^{d}$, which is dynamically replicated $N_{\mathrm{enemy}}$ times according to the current number of enemy units in the environment to form the attack action matrix. The base action embedding and the attack action embedding are concatenated to obtain a complete and adaptively adjustable action matrix $Q\in R^{(6+N_{\mathrm{enemy}})\times d}$. Subsequently, the action logits are obtained through the dot product operation $\mathrm{logits}=h\cdot Q^{T}$, where the output dimension is $R^{(6+N_{\mathrm{enemy}})\times B}$, with B denoting the batch size. Finally, unavailable actions are masked out, and a softmax function is applied to obtain the final action probability distribution.

The architecture of the Transformer-based value network is shown in the lower right part of Figure 2. The network takes the global observation as input and leverages the Transformer architecture to model variable-length observation sequences. The specific implementation is as follows: First, the global observation data is expanded into a third-order tensor and linearly mapped to a high-dimensional state space, which is then fed into the Transformer encoder layers. Subsequently, the high-dimensional state features extracted by the Transformer encoder are mean-pooled to achieve an effective representation of variable-length observations. Finally, the state value is output through a fully connected layer. This state value, together with the immediate reward from the environment, is used to compute the advantage function, which in turn guides the parameter update of the policy network.

## 5. Experiments

In this section, we will validate the cross-scenario generalization performance of LoLM-MARL in the StarCraft Multi-Agent Challenge (SMAC) environment [42]. First, we provide a brief introduction to each scenario used in the experiments. Next, we detail the baselines, experimental parameter configurations, and performance evaluation metrics. Subsequently, we evaluate the algorithm’s performance in single-task, zero-shot transfer, and few-shot transfer settings, and conduct an in-depth analysis of its cross-scenario generalization capabilities using commonly adopted core evaluation metrics for transfer learning [43]. Finally, we perform ablation studies to assess the contribution of each module to the algorithm’s overall performance.

### 5.1. Environments

The SMAC is a multi-agent reinforcement learning algorithm testing platform developed based on the real-time strategy game StarCraft II. It is one of the most widely used standard testing environments in the field of MARL. This testing environment consists of a series of collaborative multi-agent adversarial tasks, where the algorithm is required to control friendly agent combat units to engage with enemy units controlled by the built-in game AI, with the goal of achieving victory by eliminating all enemy units. Table 3 provides detailed information on the allocation of enemy and friendly combat unit numbers, as well as scenario characteristics, for both single-task and cross-scenario transfer used in the experiments. Among these, symmetric scenarios refer to scenarios where both sides have equal combat strength, while in asymmetric scenarios, there is a numerical disparity in the forces between the two sides.

### 5.2. Methods and Metric

#### 5.2.1. Baselines

To validate the advantages of the LoLM-MARL, we selected three classic cross-scenario transfer methods for comparative analysis on three types of tasks: single-task learning, zero-shot transfer, and few-shot transfer. The core concepts of each algorithm are as follows:

- DT2GS [18]: The core concept of this method lies in decomposing complex tasks into general subtasks that are independent of specific tasks. By utilizing scalable subtask encoders and adaptive subtask semantic modules, it aims to reduce the risk of overfitting to the source task and enhance the generalizability of the subtasks, thereby achieving effective cross-scenario transfer.
- ASN-Attention [44]: This method constructs a general action semantic network and decomposes the decision-making network into multiple sub-modules, each responsible for processing observation information corresponding to a specific action type, thereby eliminating interference from irrelevant information. This concept provides important insights for the development of general multi-agent decision models. For a fair comparison, we incorporate an attention mechanism into this method to enable cross-task transfer capability.
- UPDeT [45]: UPDeT decouples the observations and actions of agents, constructs specific entity-action mappings, and decomposes the policy learning task into a series of entity-centric, independently computable sub-decision modules. These modules are then flexibly coordinated through an attention mechanism to enhance adaptability to diverse tasks.

#### 5.2.2. Hyperparameter Settings

The hyperparameters used in the experiment consist of two parts: the baseline MAPPO parameter settings for LoLM-MARL and the LoRA-related parameter settings. All fixed hyperparameter configurations for the task scenarios are shown in Table 4. The pre-trained LLM employed in the experiment is Qwen3-0.6b. The number of training episodes and the annealing steps for the KL divergence are flexibly adjusted according to the difficulty level of each map. Both the representation dimensions for basic actions and attack actions are set to 1024. The number of basic actions is fixed at six, while the number of attack actions varies depending on the quantity of enemy units in the task.

All experiments were conducted on a high-performance computing server equipped with a 104-core Intel Xeon Platinum 8470Q CPU and an NVIDIA RTX PRO 6000 GPU with 96 GB memory. The software environment included PyTorch 2.7.0 and Python 3.10.18.

#### 5.2.3. Performance Metrics

To comprehensively evaluate the performance of LoLM-MARL, we adopt two types of evaluation metrics according to different evaluation objectives. Specifically, for single-task, zero-shot, and few-shot transfer experiments, we focus on the cooperative capability of the algorithm in specific tasks. For generalization performance analysis, we focus on the quality and efficiency of cross-scenario transfer. The specific metrics are as follows:

(1) Single-task, zero-shot, and few-shot transfer experiments: Following the common practice in SMAC [42], we adopt the average test win rate across multiple random seeds as the core evaluation metric to measure the cooperative performance of the algorithm in multi-agent combat tasks.

(2) Generalization performance analysis: Following the evaluation framework for transfer learning performance in reference [43], we assess the cross-scenario generalization capability of the algorithm from the following three dimensions:

- Jumpstart: the win rate at the first training timestep after transferring to the target task, reflecting the effectiveness of knowledge transfer from the source task.
- Asymptotic performance: the final win rate of the algorithm upon convergence, reflecting the upper bound of its capability.
- The number of training steps required to reach asymptotic performance: the number of interaction steps required to achieve asymptotic performance reflects the learning speed of the algorithm on the target task.

### 5.3. Performance on Single-Task

This section will provide a detailed comparative validation of LoLM-MARL’s performance on single-task learning. The experiment selects eight different scenarios covering both symmetric and asymmetric tasks, tested across three levels of difficulty: easy tasks (3s_vs_4z), hard tasks (3s5z, 8m_vs_9m, 5m_vs_6m, 10m_vs_11m, 3s_vs_5z), and super-hard tasks (3s5z_vs_3s6z, MMM2).

**Figure 5:**
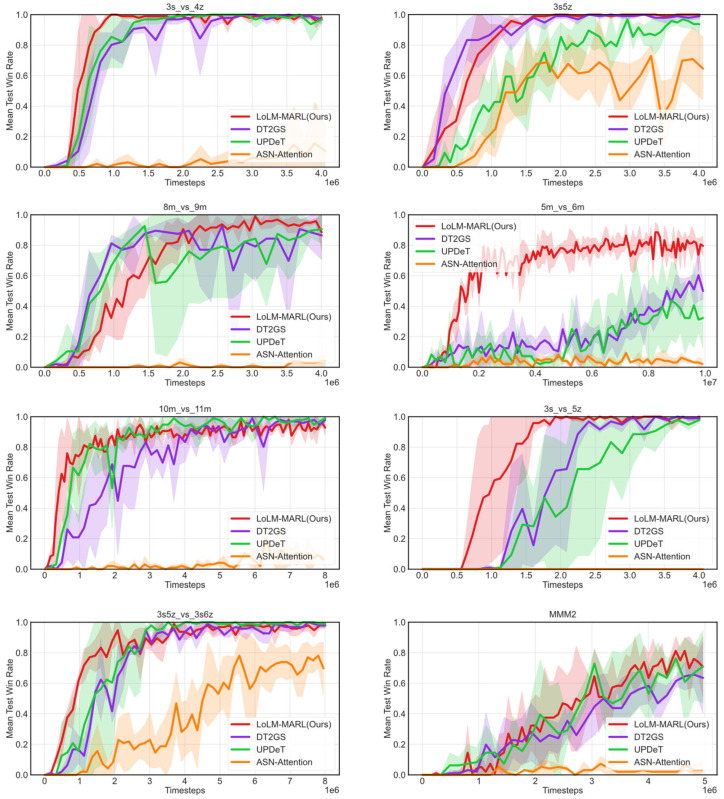
Comparative experimental results of LoLM-MARL on single-task learning.

Figure 5 presents the comparative experimental results of LoLM-MARL on single-task learning. The horizontal axis represents the number of training steps, while the vertical axis records the algorithm’s test win rate in each scenario. The red curve corresponds to the proposed method in this paper, where the solid line indicates the average of three runs with different random seeds, and the shaded area represents the standard deviation across these three runs. As shown in the figure, for the easy task 3s_vs_4z, the performance of all algorithms except ASN-Attention is relatively similar. Notably, LoLM-MARL converges around 1 × 10^6^ training steps, demonstrating significantly faster convergence compared to DT2GS and UPDeT. In hard tasks, LoLM-MARL shows absolute advantages in both asymptotic performance and convergence efficiency on 5m_vs_6m and 3s_vs_5z. For super-hard tasks, UPDeT slightly outperforms the proposed method, yet the LoLM-MARL achieves the fastest convergence speed. This indicates that the integration of LLM effectively enhances decision-making in complex MARL tasks.

### 5.4. Zero-Shot Generalization Across Scenarios

In the last section, a detailed comparative experiment and analysis were conducted on the algorithm’s collaborative performance in single tasks. This section will perform zero-shot cross-scenario transfer experiments on the policies learned by LoLM-MARL in single-task scenarios. Specifically, we deploy the collaborative policy learned by the algorithm in one scenario directly into other unseen scenarios without additional training, to verify the algorithm’s transfer reasoning capability across different scenarios. To this end, this section designs six cross-scenario zero-shot transfer experiments, namely 2s3z→1c3s5z, 3s_vs_4z→3s_vs_5z, 8m_vs_9m→5m_vs_6m, 3s5z→3s5z_vs_3s6z, 8m→10m_vs_11m and 5m_vs_6m→10m_vs_11m. These six transfer experiments cover three distinct levels of cross-scenario transfer forms. All experiments follow the design principle of transferring from a simple source task to a complex target task, with the increased complexity mainly reflected in the following three aspects: transfer from symmetric to asymmetric scenarios, an increase in the disparity between enemy and friendly forces in asymmetric scenarios, and transfer between entirely different scenarios.

Table 5 presents the comparative experimental results of zero-shot transfer. The results indicate that the proposed method possesses strong cross-scenario transfer capability, particularly evident in transfers between entirely different types of collaborative scenarios (2s3z→1c3s5z) and transfers between asymmetric challenging collaborative scenarios (8m_vs_9m→5m_vs_6m, 5m_vs_6m→10m_vs_11m).

**Figure 6:**
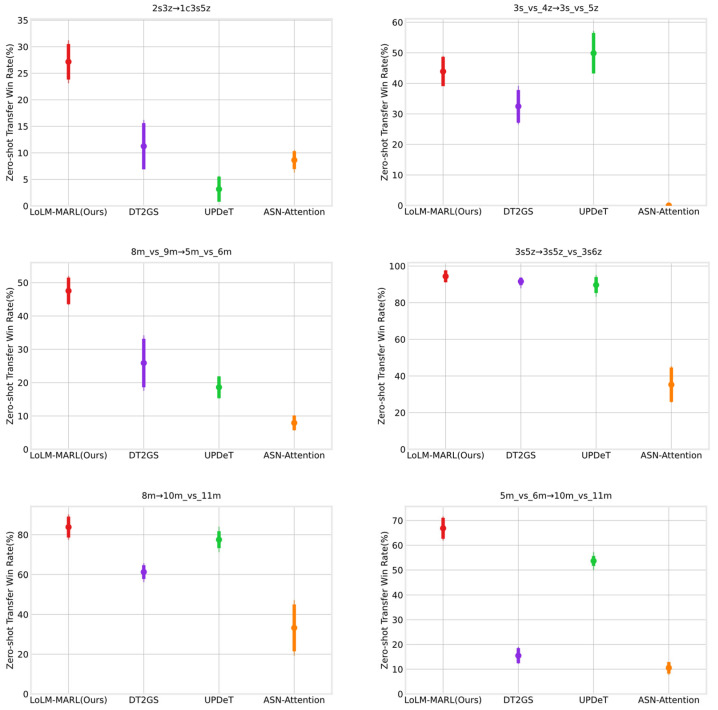
Visualized comparison results of zero-shot transfer for each algorithm. Dots represent the average win rate, thick vertical bars indicate the standard deviation of the data, and thin vertical bars show the extreme values.

In contrast, in transfer task 2s3z→1c3s5z, UPDeT shows weaker adaptability to completely unfamiliar scenarios, while LoLM-MARL achieves a transfer win rate on the target task that is twice that of the suboptimal algorithm DT2GS. This indicates that the proposed method can learn more general and transferable policies from the source task, exhibiting a lower rate of knowledge forgetting compared to other algorithms. For transfers between asymmetric challenging collaborative scenarios, Figure 6 clearly illustrates the significant advantages of LoLM-MARL in transfer tasks 8m_vs_9m→5m_vs_6m and 5m_vs_6m→10m_vs_11m. In this figure, dots represent the average win rate, thick vertical bars indicate the standard deviation of the data, and thin vertical bars show the extreme values. It can also be observed from the figure that the ASN-Attention generally exhibits weak cross-scenario transfer capability. Combining with the performance of each algorithm in single-task scenarios shown in Figure 5, a conclusion can be drawn: strong collaborative capability in a single task is a crucial foundation for cross-scenario transfer. If an algorithm fails to learn robust and effective cooperative policies in a single task, it is also unlikely to achieve stable transfer performance during cross-scenario adaptation.

### 5.5. Few-Shot Generalization Across Scenarios

In Section 5.4, extensive experiments were conducted to validate the zero-shot cross-scenario transfer performance of LoLM-MARL. To further verify the cross-scenario transfer capability of the proposed method under few-shot conditions, this section similarly follows the experimental design of the previous section to carry out comparative experiments across six cross-scenario tasks. Different from the experimental setup in the previous section, few-shot cross-scenario transfer refers to fine-tuning the parameters of the algorithm trained on the source task using a small number of samples in the target task, thereby enabling the algorithm to quickly adapt to the new scenario. Due to the varying difficulty levels of the tasks, the number of training timesteps for each transfer task is not exactly the same. Based on the convergence observed in multiple cross-validation experiments of the algorithm, this section sets the interaction timesteps for the four tasks 2s3z→1c3s5z, 8m_vs_9m→5m_vs_6m, 3s5z→3s5z_vs_3s6z and 8m→10m_vs_11m to 3 × 10^5^, for task 3s_vs_4z→3s_vs_5z to 1 × 10^6^, and for task 5m_vs_6m→10m_vs_11m to 6 × 10^5^.

**Figure 7:**
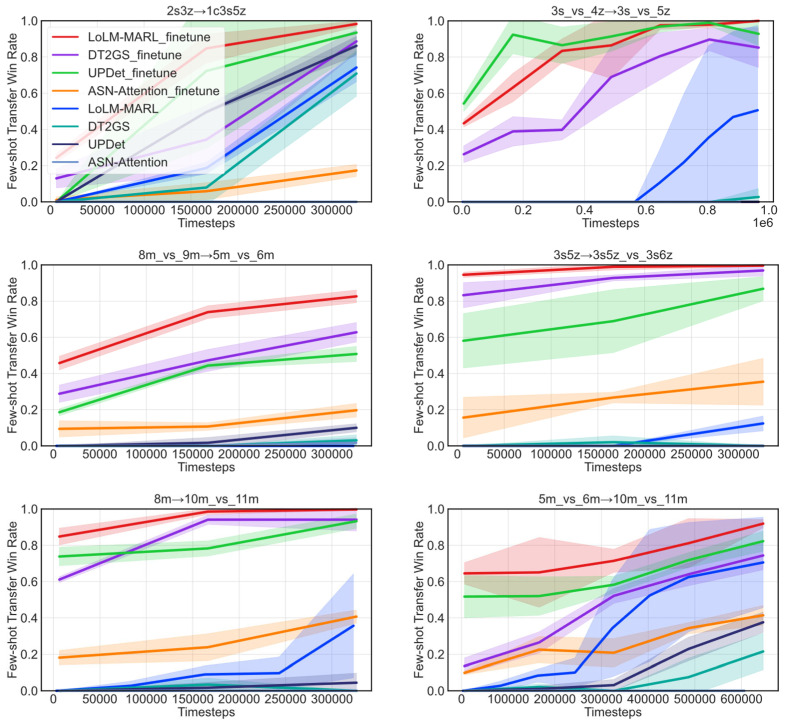
Comparative experimental results of few-shot transfer for each algorithm.

Figure 7 presents the transfer experimental results of the fine-tuned algorithms and their corresponding original algorithms under few-shot data. Overall, LoLM-MARL demonstrates clear advantages in each of the few-shot transfer tasks. Specifically, it achieves win-rate advantages of approximately 20% and 10% over the suboptimal methods in transfer tasks 8m_vs_9m→5m_vs_6m and 5m_vs_6m→10m_vs_11m. Meanwhile, LoLM-MARL attains nearly 100% win rates in the other transfer tasks, effectively illustrating the generalization capability of LLM-based multi-agent reinforcement learning in cross-scenario transfer. In contrast, ASN-Attention struggles to achieve effective transfer to unseen scenarios. We analyze that a possible reason is the distributional shift in task semantics during scenario switching. Relying solely on predefined static primitive action semantics fails to adequately match the action requirements of new tasks, thereby limiting the algorithm’s transfer capability.

The learning speed of an algorithm on the target task largely reflects its transfer capability. How to effectively and rapidly apply knowledge learned from the source task to the target task is one of the core metrics for evaluating algorithm transfer performance. Based on the experimental results shown in Figure 7, it can be observed that LoLM-MARL_finetune achieves a high win rate within only 6400 training steps, particularly in transfer tasks 3s5z→3s5z_vs_3s6z and 8m→10m_vs_11m, where it nearly reaches the asymptotic performance of algorithmic convergence. This clearly demonstrates that the fine-tuning approach can fully leverage prior knowledge acquired from the source task to enable rapid learning in the target task.

To demonstrate the necessity and effectiveness of cross-scenario transfer research, we also compared the performance of the original algorithm and the fine-tuned algorithm on the target task. As shown in Figure 7, in most test scenarios, only the original LoLM-MARL algorithm achieved a certain win rate within limited training steps, but its stability (shaded area in the figure) was significantly weaker than that of the fine-tuning-based method (LoLM-MARL_finetune). This indicates that without guidance from prior knowledge of the source task, the model struggles to learn effective and stable cooperative policies in a short time. In contrast, the fine-tuning method introduces collaborative experience learned from the source task, providing the model with reasonable initialization directions. This approach significantly reduces the policy exploration space and accelerates the convergence speed of the algorithm on the target task.

### 5.6. Generalization Analysis

Evaluating the cross-scenario generalization performance of MARL primarily involves two aspects: first, whether the algorithm can leverage policies learned from the source task to guide its learning in similar but distinct target tasks; and second, how well the algorithm utilizes existing knowledge. This section will address these two questions and systematically conduct an in-depth analysis of the generalization capability of LoLM-MARL by integrating performance metrics [42] from transfer reinforcement learning. The analysis will proceed in two steps: first, by comparing the performance of the fine-tuned algorithm LoLM-MARL_finetune and the original algorithm LoLM-MARL on the target task to verify whether the algorithm possesses cross-scenario generalization ability; and second, by comparing the LoLM-MARL_finetune algorithm with other transfer algorithms to demonstrate the generalization capability of the proposed method.

Regarding the first question, we compare and analyze the performance of the fine-tuning method and the original learning-from-scratch method on the target task. Based on transfer learning performance metrics, this section focuses on three commonly used indicators: jumpstart, asymptotic performance, and the number of training steps required to reach asymptotic performance. Table 6 details the experimental results across these metrics. It can be seen that the learning-from-scratch method LoLM-MARL exhibits almost no learning capability in the early training stage. In contrast, the fine-tuned algorithm LoLM-MARL_finetune demonstrates higher initial learning performance, which helps the algorithm explore a more optimal policy space and provides important support for rapid convergence on unseen tasks. In terms of asymptotic performance, LoLM-MARL_finetune achieves slight improvements across all six transfer tasks. We analyze the possible reasons as follows: the integration of LLM and MARL significantly enhances LoLM-MARL’s performance in single-task scenarios, nearly achieving the optimal test win rate. Therefore, in terms of algorithm limitations, the single-task performance itself already approaches the theoretical upper limit of the environment, leaving relatively limited room for potential improvement through transfer learning. Regarding asymptotic convergence speed, Table 6 shows that LoLM-MARL_finetune achieves a 4–30× speedup, primarily attributed to the general decision-making knowledge learned by the LLM from the source task. This provides an effective initial exploration policy for the algorithm, significantly reducing exploration costs in the target task and minimizing the probability of random and ineffective exploration during the early learning stage.

**Figure 8:**
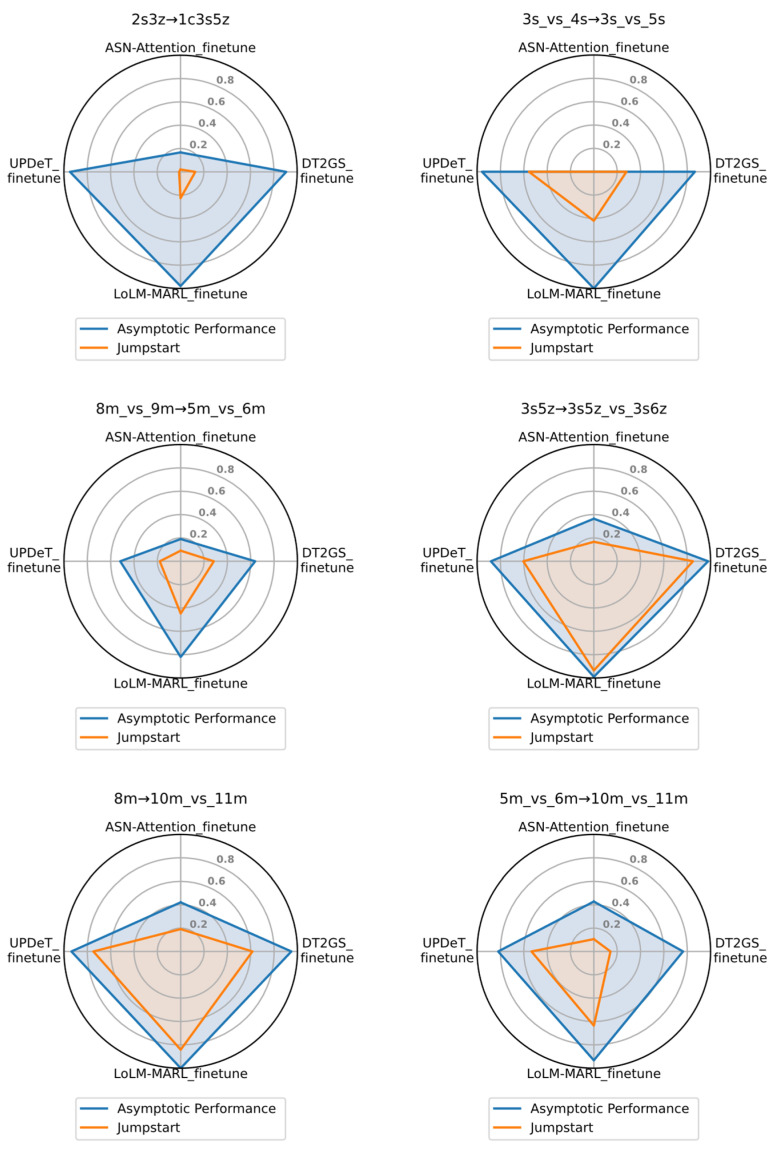
Comparison results of jumpstart and asymptotic performance of the fine-tuning method across six types of transfer tasks.

Next, we will explore the cross-scenario generalization capability of the proposed method by comparing it with other transfer methods. Figure 8 illustrates the transfer performance of four methods on the target task, where the red line represents jumpstart on the target task, and the blue line represents the asymptotic performance. As can be clearly seen from the figure, except for a slightly lower jumpstart than the UPDeT_finetune algorithm in the transfer task 3s_vs_4z→3s_vs_5z, LoLM-MARL_finetune demonstrates significant advantages in both initial and asymptotic performance across all other transfer tasks. It is worth noting that although UPDeT_finetune outperforms LoLM-MARL_finetune in initial performance in transfer task 3s_vs_4z→3s_vs_5z, its asymptotic performance falls short of the proposed method. This indirectly reflects the learning potential of our method in cross-scenario transfer tasks. In terms of convergence speed, based on the few-shot transfer experimental results in Figure 7, the proposed method exhibits faster convergence, with the advantages being more pronounced in challenging tasks. Furthermore, combining the visual results from Figure 8 reveals that jumpstart performance serves as the foundation for asymptotic performance, with a clear positive correlation between the two. Strong initial learning performance helps the algorithm explore more promising solution spaces early in training, facilitating subsequent cumulative reward improvement and final policy convergence.

### 5.7. Ablation Studies

To thoroughly validate the effectiveness of each module in LoLM-MARL, this section conducts ablation analyses on the LLM dynamic prompt module and the KL divergence regularization module based on the annealing strategy. For the dynamic prompt module, we primarily analyze its impact on transfer performance; for the KL divergence regularization module, the focus is on its influence on stability during source task training.

**Figure 9:**
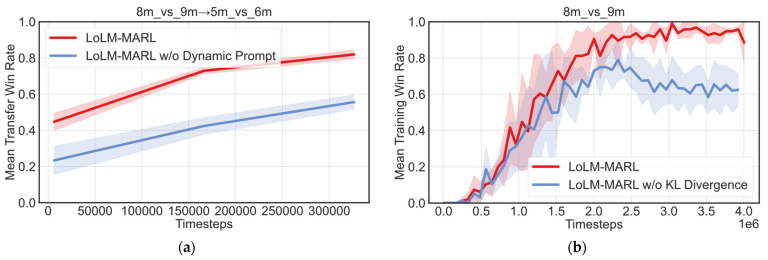
Ablation studies results of each module. (**a**) Impact of dynamic prompts on transfer performance; (**b**) impact of KL divergence on source task training stability.

Taking transfer task 8m_vs_9m→5m_vs_6m as an example, Figure 9a presents a comparison of transfer performance on the target task between LoLM-MARL and LoLM-MARL w/o dynamic prompt, which employs a fixed prompt scheme. The figure clearly shows that the dynamic prompt design significantly improves both the initial performance and the asymptotic performance of the algorithm on the target task, while also conferring higher transfer stability. This indicates that dynamic prompts enable the algorithm to cover a wider variety of training scenarios during source task learning, thereby facilitating the acquisition of more general collaborative policies and ultimately enhancing its cross-scenario generalization capability.

The ablation experimental results for KL divergence are shown in Figure 9b. It can be observed that LoLM-MARL w/o KL Divergence exhibits a performance decline after approximately 2.35 × 10^6^ training steps. This is due to the lack of KL divergence constraints during the early stages of LoRA fine-tuning, which causes the fine-tuned model to deviate too rapidly from the prior distribution of the pre-trained LLM. This increases the randomness of policy exploration, leads the algorithm to converge prematurely to suboptimal solutions, and gradually manifests catastrophic forgetting as training progresses. In contrast, the LoLM-MARL leverages KL divergence to regularize the fine-tuned LLM, ensuring that the policy distribution remains aligned with that of the pre-trained LLM during the initial stage of fine-tuning. This guides the algorithm toward exploring a more optimal policy space.

**Figure 10:**
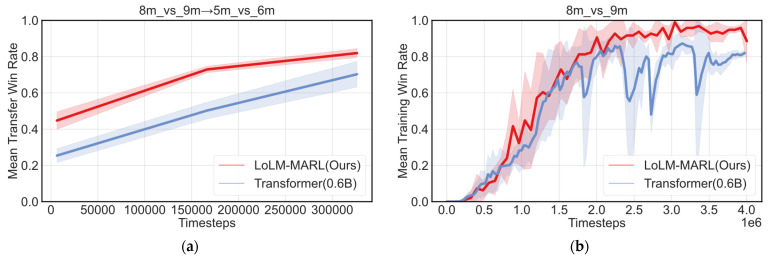
Ablation study results of the policy network baseline. (**a**) Comparison of transfer performance between Transformer (0.6B) and LoLM-MARL; (**b**) comparison of experimental results between Transformer (0.6B) and LoLM-MARL on the source task.

To further verify that the improvements of LoLM-MARL in single-task performance and cross-scenario generalization capability are mainly attributed to the pre-trained prior knowledge and reasoning abilities of the LLM, rather than the gain from parameter scaling, we designed an ablation experiment: replacing the pre-trained LLM in the policy network with a randomly initialized Transformer model (0.6B) of the same parameter scale. The experimental results are shown in Figure 10.

Figure 10a presents the comparative transfer experiment results between the randomly initialized Transformer model (0.6B) and LoLM-MARL. It can be observed that LoLM-MARL outperforms Transformer (0.6B) in both initial performance and convergence performance, fully demonstrating that the cross-scenario generalization capability of LoLM-MARL is not merely a result of increasing model parameters. Figure 10b also shows the performance comparison results on the single-task setting. It can be observed that the algorithm using Transformer (0.6B) as the baseline performs relatively poorly. Around 1.75 × 10^6^ training steps, it exhibits significant fluctuations, and its final convergence performance is substantially lower than that of LoLM-MARL. These results further demonstrate that the performance advantage of the proposed method on single tasks mainly stems from the pre-trained prior knowledge and reasoning capabilities of the LLM.

To more thoroughly examine the influence of the LLM on the exploration mechanism in MARL, we compared the policy entropy of the vanilla MAPPO algorithm and the LLM-assisted LoLM-MARL algorithm during training on task 8m_vs_9m, thereby providing deeper insight into how the LLM affects collaborative agent behavior.

**Figure 11:**
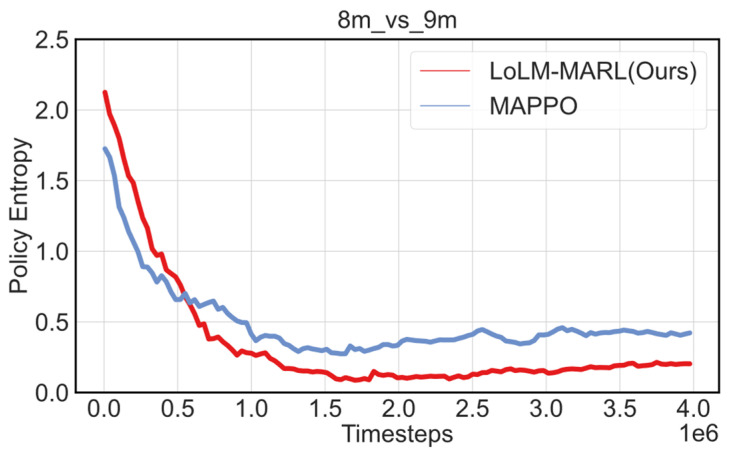
Ablation study results of policy entropy.

As shown in Figure 11, in the early stage of policy exploration, the policy entropy of LoLM-MARL is significantly higher than that of MAPPO, indicating that the prior knowledge of the LLM does not force the agent to prematurely converge to a deterministic policy. Instead, it encourages more diverse initial exploration, which helps the agent discover better policy spaces in the early stage. As training progresses, the policy entropy of LoLM-MARL rapidly decreases and stabilizes at a low level, while the entropy of MAPPO decreases more slowly and remains at a relatively high level. This demonstrates that LoLM-MARL can converge more quickly to deterministic policies after sufficient exploration, reflecting the positive role of the LLM in balancing exploration and exploitation. Consequently, this promotes rapid convergence of the algorithm and enhances its ability to explore optimal policies.

## 6. Conclusions

In this work, we propose a MARL policy transfer method, LoLM-MARL, based on large language model fine-tuning to solve the problem that the traditional MARL algorithm is difficult to generalize when facing complex decision tasks. This method leverages lightweight and efficient parameter fine-tuning through LoRA, combined with the rich prior knowledge and strong reasoning capabilities of LLMs, to realize a semantics-driven multi-agent collaborative decision-making scheme. Moreover, to further enhance the adaptability of LLMs in specific collaborative decision-making tasks, we skillfully designed a dynamic prompt construction method that provides LLMs with broader training scenarios and denser state information, effectively ensuring few-shot and zero-shot transfer capabilities across scenarios. Additionally, to mitigate the potential catastrophic forgetting problem during the early stages of fine-tuning, this paper introduces a KL divergence regularization method based on an annealing strategy, which dynamically constrains the action probability distributions of both the pre-trained model and the fine-tuned model. This work represents an exploratory attempt at the intersection of LLMs and MARL. Extensive experimental results demonstrate that, across similar but not identical scenarios, the proposed method achieves significantly superior generalization performance in both zero-shot and few-shot transfer tasks compared to traditional SOTA methods, thereby providing a new technical pathway for reducing the deployment cost of multi-agent systems in real-world settings and accelerating the rapid adaptation of agents to unseen environments.

### Limitations and Future Work

Although LoLM-MARL outperforms traditional methods in terms of generalization performance, it still has the following limitations. First, due to the large number of parameters of the LLM, even with LoRA fine-tuning, its training and inference overhead remain significantly higher than those of traditional MARL methods, making it difficult to directly apply to scenarios with high real-time requirements. This, to some extent, limits its applicability in resource-constrained environments. Second, LoLM-MARL requires manually designing semantic mapping rules from numerical observations to natural language. When switching to a completely different task for retraining (e.g., from StarCraft to MPE), the prompt template needs to be redesigned according to the new task and environment characteristics, thereby increasing the complexity of algorithm design.

To address the above limitations, future work will focus on two main aspects. First, we will explore lightweight LLM backbones and inference acceleration strategies, such as model compression or sparse attention mechanisms, to reduce computational overhead and meet the requirements of real-time applications. Second, we will investigate automated generation methods for prompt templates to reduce the manual design cost caused by task switching, thereby improving the generalization and deployment efficiency of the algorithm across different environments.

## Figures and Tables

**Table 1:** Composition of raw observation space for SMAC.

Observed Entities	Observation Space
Ally	Visible/Not visible
	Distance
	Relative position along the X-axis
	Relative position along the Y-axis
	Health
	Shield
	Unit type
	Last action
Enemy	Attack availability
	Distance
	Relative position along the X-axis
	Relative position along the Y-axis
	Health
	Shield
	Unit type
Self	Health
	Shield
	Unit type
	Last action
	Agent ID

**Table 2:** Composition of primitive action space for SMAC.

Action	Description
No Operation	Do nothing (only applicable to dead agents)
Stop	Stop all actions: the agent remains in stationary state
Move Direction	Move a fixed distance in four directions (North, South, East, and West)
Attack ID	Attack the enemy with the specified ID

**Table 3:** Configuration information for each scenario in SMAC.

Scenario	Ally	Enemy	Scenario Characteristics
3s5z	3 Stalkers & 5 Zealots	3 Stalkers & 5 Zealots	Symmetric
8m_vs_9m	8 Marines	9 Marines	Asymmetric
5m_vs_6m	5 Marines	6 Marines	Asymmetric
10m_vs_11m	10 Marines	11 Marines	Asymmetric
3s_vs_4z	3 Stalkers	4 Zealots	Asymmetric
3s_vs_5z	3 Stalkers	5 Zealots	Asymmetric
3s5z_vs_3s6z	3 Stalkers & 5 Zealots	3 Stalkers & 6 Zealots	Asymmetric
MMM2	1 Medivac, 2 Marauders & 7 Marines	1 Medivac, 3 Marauders & 8 Marines	Asymmetric
2s3z	2 Stalkers & 3 Zealots	2 Stalkers & 3 Zealots	Symmetric
1c3s5z	1 Colossus, 3 Stalkers & 5 Zealots	1 Colossus, 3 Stalkers & 5 Zealots	Symmetric
8m	8 Marines	8 Marines	Symmetric

**Table 4:** Experimental hyperparameter settings.

Hyper-Parameters	Value
Discount factor	0.99
Prompt batch size	64
LoRA learning rate	5 × 10^−6^
Action head learning rate	5 × 10^−5^
Value network learning rate	5 × 10^−5^
Maximum episode length	200
LoRA rank	8
LoRA scaling coefficient	16
LoRA dropout	0.1
LoRA target modules	q, k, v, o attention projection layer
Number of value network Transformer encoder layers	1
Number of self-attention heads in Transformer encoder	4
$\lambda_{0}$	20

**Table 5:** Zero-shot transfer win rate comparison results.

Transfer Task	DT2GS	UPDeT	ASN-Attention	LoLM-MARL (Ours)
2s3z→1c3s5z	11.25 ± 4.36	3.15 ± 2.37	8.63 ± 1.69	**27.17 ± 3.35**
3s_vs_4z→3s_vs_5z	32.46 ± 5.36	**49.86 ± 6.64**	0.00 ± 0.00	43.89 ± 4.79
8m_vs_9m→5m_vs_6m	25.89 ± 7.28	18.62 ± 3.26	7.93 ± 2.21	**47.54 ± 3.98**
3s5z→3s5z_vs_3s6z	91.59 ± 2.06	89.64 ± 4.36	35.26 ± 9.38	**94.38 ± 3.25**
8m→10m_vs_11m	61.25 ± 3.42	77.52 ± 4.26	33.26 ± 11.78	**83.84 ± 5.27**
5m_vs_6m→10m_vs_11m	15.48 ± 3.06	53.67 ± 2.02	10.56 ± 2.35	**66.87 ± 4.23**

**Table 6:** Performance comparison results between the fine-tuning method LoLM-MARL_finetune and the original method LoLM-MARL on the target task.

Transfer Task	Jumpstart (%)		Asymptotic Performance (%)		The Training Step Required for Asymptotic Performance	
	LoLM-MARL	LoLM-MARL Finetune	LoLM-MARL	LoLM-MARL Finetune	LoLM-MARL	LoLM-MARL Finetune
2s3z→1c3s5z	0.00 ± 0.00	22.62 ± 2.55	97.64 ± 1.50	97.92 ± 1.47	4 × 10^6^	3.26 × 10^5^
3s_vs_4z→3s_vs_5z	0.00 ± 0.00	42.04 ± 2.86	1.00 ± 0.00	1.00 ± 0.00	4 × 10^6^	1 × 10^6^
8m_vs_9m→5m_vs_6m	0.00 ± 0.00	44.75 ± 3.95	79.54 ± 5.48	81.96 ± 2.04	1 × 10^7^	3.26 × 10^5^
3s5z→3s5z_vs_3s6z	0.00 ± 0.00	93.54 ± 2.30	97.92 ± 1.47	98.96 ± 1.47	8 × 10^6^	3.26 × 10^5^
8m→10m_vs_11m	0.00 ± 0.00	84.17 ± 3.08	92.71 ± 3.90	1.00 ± 0.00	8 × 10^6^	3.26 × 10^5^
5m_vs_6m→10m_vs_11m	0.00 ± 0.00	63.54 ± 5.91	92.71 ± 3.90	93.29 ± 3.03	8 × 10^6^	6.46 × 10^5^

## Data Availability

The data presented in this work are available on request from the corresponding authors.

## Appendix A

The complete semantic information of the dynamic prompt in Figure 4 is shown in Table A1 and Table A2. From the information in the Tables combined with Figure 4, it can be seen that the information of dead agents is promptly filtered out. This design philosophy of prompt construction effectively reduces the interference of redundant information on LLM decision-making and improves its decision-making capability in specific scenarios.

**Table A1:** Semantic information of agent 0′s observations of allies.

Entity	Visibility	Type	Distance	Relative Position	Health	Shield	Last Action
Ally 1	is visible	stalker	0.39	(0.31, −0.23)	1.00	1.00	attack enemy 3
Ally 2	is visible	stalker	0.85	(0.67, −0.53)	1.00	1.00	move south
Ally 3	is visible	zealot	0.96	(0.81, 0.51)	0.80	0.00	attack enemy 1
Ally 4	is visible	zealot	1.24	(1.16, −0.43)	0.60	0.00	move north
Ally 5	is visible	zealot	1.25	(1.18, 0.40)	1.00	0.60	move west
Ally 6	is visible	zealot	1.04	(0.95, 0.42)	0.80	0.00	attack enemy 1
Ally 7	is visible	zealot	1.17	(1.13, 0.32)	1.00	0.20	move east

**Table A2:** Semantic information of agent 0′s observations of enemies.

Entity	Attackable	Type	Distance	Relative Position	Health	Shield
Enemy 0	is available attack	stalker	1.99	(1.79, 0.87)	1.00	1.00
Enemy 1	is available attack	stalker	1.15	(0.92, 0.69)	1.00	0.29
Enemy 2	is available attack	stalker	1.48	(1.40, 0.48)	1.00	1.00
Enemy 3	is available attack	zealot	1.37	(1.25, 0.57)	1.00	0.80
Enemy 4	is available attack	zealot	1.35	(1.01, 0.90)	1.00	1.00
Enemy 6	is available attack	zealot	1.69	(1.51, 0.76)	1.00	1.00
